# Laboratory Operando XAS Study of Sodium Iron Titanite Cathode in the Li-Ion Half-Cell

**DOI:** 10.3390/nano11010156

**Published:** 2021-01-09

**Authors:** Victor Shapovalov, Alexander Guda, Vera Butova, Igor Shukaev, Alexander Soldatov

**Affiliations:** 1 The Smart Materials Research Institute, Southern Federal University, 178/24 A. Sladkova Street, 344090 Rostov-on-Don, Russia; guda@sfedu.ru (A.G.); vbutova@sfedu.ru (V.B.); 2 Department of Chemistry, Southern Federal University, 7 Zorge Street, 344090 Rostov-on-Don, Russia; ishukaev@sfedu.ru

**Keywords:** Na-ion, cathode material, sodium iron titanate, operando study, X-ray absorption spectroscopy, DFT calculations

## Abstract

Electrochemical characterization of the novel sodium iron titanate Na_0.9_Fe_0.45_Ti_1.55_O_4_ was performed upon cycling in the Li-ion half-cell. The material exhibited stable cycling in the voltage range 2–4.5 V, and the number of alkali ions extracted per formula unit was approximately half of the Na stoichiometry value. Using laboratory X-ray absorption spectrometry, we measured operando Fe K-edge X-ray absorption spectra in the first 10 charge–discharge cycles and quantified the portion of charge associated with the transition metal redox reaction. Although 3d metals are commonly accepted redox-active centers in the intercalation process, we found that in all cycles the amount of oxidized and reduced Fe ions was almost 20% less than the total number of transferred electrons. Using density functional theory (DFT) simulations, we show that part of the reversible capacity is related to the redox reaction on oxygen ions.

## 1. Introduction

The trend in modern energetics toward increasing the use of renewable power sources heavily depends on the development of efficient energy storage and conversion systems [1,2,3]. In this scope, Na-ion batteries have drawn increased attention for their applications in large-scale energy storage facilities [4]. Much effort has been invested in the development of hybrid Li/Na-ion systems based on polycationic electrode materials or the use of Li-based (Na-based) electrode or electrolyte in Na-based (Li-based) electrochemical systems. Such combinations may benefit from both types of alkali metal chemistries and compensate for their primary drawbacks [5]. As an example, the electrochemical performance of overlithiated oxide cathode materials may be drastically improved by concentration-difference-driven molten salt Li/Na ion exchange [6]. Another promising class of materials is Na-rich rocksalt oxides, Na_1 + x_M_1 − x_O_2_ (M = transition metal), combining cationic and anionic redox activity. Their synthesis remains challenging, and mixed compounds such as Na(A_x_M_1 − x_)O_2_ (A = Li, Mg, or Zn) may become an alternative. However, it is challenging to achieve the stability of such materials in both Li and Na systems [7]. Another step toward dual-ion systems with improved performance may be the employment of materials with mixed d-metal cations or materials that exhibit anion redox reactions such as oxygen redox. In this scope, Ti-doped polyanionic or Na super-ionic conductor (NASICON) type electrodes, as well as pure titanate-based cathodes, have become a hot topic [8]. Carbon coating increases the capacity and improves the high-rate stability of LiTi_2_(PO_4_)_3_ in aprotic Na cells [9]. Density functional theory (DFT) calculations show that the electrochemical ion exchange of Li ions by Na during cycling occurs through a two-phase reaction.

The processes in electrochemical systems are complex and require a deep understanding of the chemical composition as well as atomic and electronic structure of electrode materials, and the dynamics of their change during battery operation [10,11]. Operando techniques provide the most reliable and authentic insight into the internal electrochemical reactions of the cell, including transient processes and short-living intermediate species [12,13,14]. Recently there were several works published on the study of advanced polyanionic, Na-rich, and NASICON-type electrode materials for Na-ion batteries [15,16]. Broux et al. reported the V disproportionation upon sodium extraction confirmed for Na_3_V_2_(PO_4_)_2_F_3_ polyanionic cathode utilizing operando X-ray absorption spectroscopy (XAS) [17]. Principal component analysis (PCA) of V K-edge X-ray absorption near-edge structure (XANES) data enabled the extraction of three components, corresponding to the initial V^III^-V^III^, intermediate V^III^–V^IV^, and final V^III^–V^V^ phases. NASICON-type Na_3_VCr(PO_4_)_3_ was investigated by in situ synchrotron X-ray diffraction (XRD) and ex situ XAS and nuclear magnetic resonance (NMR) spectroscopy [18], which revealed V^3+^/V^4+^ and V^4+^/V^5+^ redox couples, and the mechanism of the capacity decay was explained by the structural irreversibility with the formation of a metastable phase [19]. A combination of in situ XRD, operando transmission X-ray microscopy, and XANES (TXM-XANES) was used to unravel the origin of irreversible capacity loss in sodium metal-oxides [20]. It was reported that at the single-particle level, the electrochemically active phase transformation exhibits a dissymmetric spatial distribution with a “core–shell” reaction mechanism, which exhibits a different discharge behavior due to the existence of the irreversible phase (Na_0.17_NiO_2_). Combined experimental and theoretical investigation of a novel NASICON-type Na_3.5_Mn_0.5_V_1.5_(PO_4_)_3_ cathode was reported by Zhang et al [21]. In situ XRD revealed that the electrochemical process exhibited highly reversible biphasic transition. The Mn^2+^/Mn^3+^ and V^3+^/V^4+^ redox couples occurring during the Na^+^ extraction/insertion were further verified by ex situ XANES and X-ray photoelectron spectroscopy (XPS) spectra. DFT computations revealed that the intrinsic low Na^+^ migration energy barrier was responsible for the superb rate capability of this material. Ti-doped NASICON-Na_3_VTi(PO_4_)_3_ cathodes were investigated by means of in situ synchrotron XRD and XAS to unravel the underlying sodium storage mechanism and charge compensation behavior [22]. Analysis of V and Ti charge states addressed by XANES showed that initial charge may be attributed to V^3+^/V^4+^ oxidation, while Ti^3+^/Ti^4+^ reduction occurs on discharge.

Operando research of battery materials usually implies the use of synchrotron radiation due to its high flux, allowing better time and spatial resolution, and the ability to maintain a proper sample environment for moisture- and oxygen-sensitive materials through the use of special electrochemical cells or chambers. However, the use of carefully tuned equipment and compatible, thoroughly designed, X-ray-transparent electrochemical cell allows one to perform operando experiments using only laboratory equipment. Recently, laboratory XAS and Mössbauer spectroscopy were applied in an operando regime to address the details of redox processes in a series of Li-ion cathodes based on LiFe_0.5_Mn_0.5_PO_4_ mixed d-metal olivines [23]. In the present work, we studied a novel Na-ion cathode material based on ${\mathrm{Na}}_{x}{\mathrm{Fe}}_{\frac{x}{2}}{\mathrm{Ti}}_{2-\frac{x}{2}}O_{4}$ sodium iron titanate and its electrochemical performance in a Li-ion half-cell. Preliminary results of electrochemical cycling suggest that samples with x = 0.9 show promising electrochemical performance, including high stability for demanding systems such as dual Li/Na-ion batteries. The local atomic and electronic structure of this material was investigated by means of laboratory X-ray absorption spectroscopy in an operando regime during charge and discharge. Theoretical ab initio DFT calculations were applied to investigate the peculiarities of redox processes in the dual-ion system.

## 2. Materials and Methods

The synthesis was performed according to the protocol described in [24]. Starting materials included FeTiO_3_, sodium carbonate (Na_2_CO_3_), and rutile (TiO_2_). TiO_2_ and Na_2_CO_3_ were calcinated for purifying. In the first stage, we obtained an intermediate compound—Na_8_Ti_5_O_14_—using sodium carbonate and TiO_2_ in the solid-state reaction [24]. In the second stage, fine powders of FeTiO_3_, TiO_2_, and Na_8_Ti_5_O_14_ were mixed in the molar ratio 4:4.78:1, and ground and pressed into a pellet. The pellet was heated at 1050 °C in a tube oven in argon flow for 6 h. After synthesis, the sample was rapidly cooled to prevent possible phase changes. To obtain a fully oxidized phase, the sample of Na_0.9_Fe_0.45_Ti_1.55_O_4_ was calcined for 5 h in air at 1000 °C. After this treatment, the color was changed from black to brown. It was designated as Ox-Na_0.9_Fe_0.45_Ti_1.55_O_4_.

Powder X-ray diffraction (XRD) profiles were recorded on a D2 Phaser laboratory X-ray diffractometer (Bruker Corporation, Billerica, MA, USA) in the 2θ range from 10° to 60° using Cu Kα radiation (1.54056 Å) with a step of 0.01°. Profile analysis was performed using the Jana2006 program (Laboratory of Crystallography, Institute of Physics, Prague, Czech Republic) [25].

X-ray absorption near edge structure (XANES) spectra were measured in operando conditions with the R-XAS Looper (Rigaku, Tokyo, Japan) laboratory X-ray absorption spectrometer at the Smart Materials Research Institute of Southern Federal University. All measurements were performed in transmission geometry with Ge (311) crystal as a monochromator, providing energy resolution ΔE = 1.4 eV for the Fe K-edge. The incident beam intensity was measured by Ar-filled (300 mbar) ionization chamber, and the transmitted intensity by a scintillation counter with a photomultiplier tube. Acquired spectra were normalized and flattened using the Athena tool from the Demeter package [26]. No energy correction or averaging of operando data were performed. Spectra for reference compounds were measured in 4 scans with subsequent averaging, normalization, and flattening. PCA of operando data was performed using PyFitIt software (v. 2.0.7, The Smart Materials Research Institute, Southern Federal University, Rostov-on-Don, Russia) [27,28].

**Figure 1:**
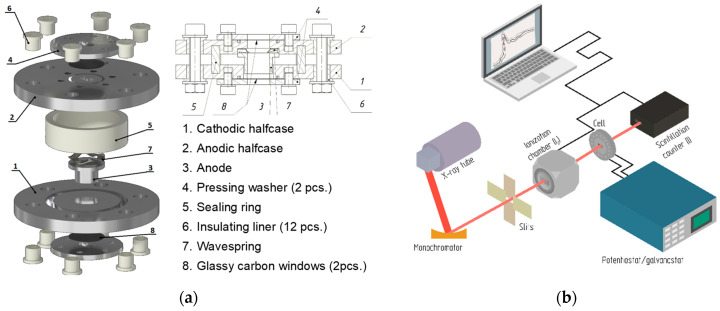
(**a**) Design of the electrochemical cell for laboratory operando XAS studies. (**b**) Scheme of the experimental setup. The main parts of the laboratory X-ray absorption spectrometer are shown, the cell is installed on the sample position and connected to a potentiostat/galvanostat device.

A home-built electrochemical cell [23] was used to enable operando XAS measurements in a transmission geometry (Figure 1). The cell is equipped with a set of large glassy carbon (GC) windows to allow X-rays to pass through the sample inside the cell. The cathode material is placed on one of the windows, which acts as a current collector. The cathode sample and anode foil are separated by Whatman grade 70 glass fiber filter and pressed together using a spring-loaded copper anode current collector, thus providing optimal electrochemical performance for a varying amount of cathode material. The cell’s design allows for adjustment of the volume of cathode sample to achieve an optimal value of the total sample absorption, absorbance edge step, and signal-to-noise ratio.

Sample powder was mixed and mortared with 20 wt% of carbon (Timcal Super P Conductive, Alpha Aesar) and placed as a cathode into the electrochemical cell with a load of 13.7 mg/cm^2^ (calculated for optimal total absorption and edge-step using XAFSmass software (v. 1.4.0, Konstantin Klementiev, MAX IV Laboratory, Lund, Sweden) [29]. Li foil was used as an anode and 1 M LiPF_6_ in 1:1 EC:DMC commercial solution (Sigma) was used as an electrolyte. The cell was cycled in the 1.6 to 4.5 V range with a current of C/20 mA (where C is the theoretical cell capacity).

Ab initio DFT calculations were performed using the VASP package (v. 5.4, VASP Software GmbH, Vienna, Austria) [30,31] with a set of GGA-PBE projector-augmented-wave pseudopotentials with spin polarization. GGA + U correction was added according to the Dudarev approach with (U-J) = 7 eV for both Fe and Ti atoms. For each stoichiometry of alkali metal, a two-step geometry optimization was performed, including cell volume and then atomic positions. The conjugate gradient algorithm was used for ionic relaxation and a special Davidson block iteration scheme for electronic minimization. Optimized structures were further used for accurate single-point calculations of total energy, charge densities, and atomic charges according to the Bader method [32,33]. The same calculation steps were repeated to obtain Bader charge values from various reference compounds with known Fe and Ti oxidation states.

## 3. Results and Discussion

**Figure 2:**
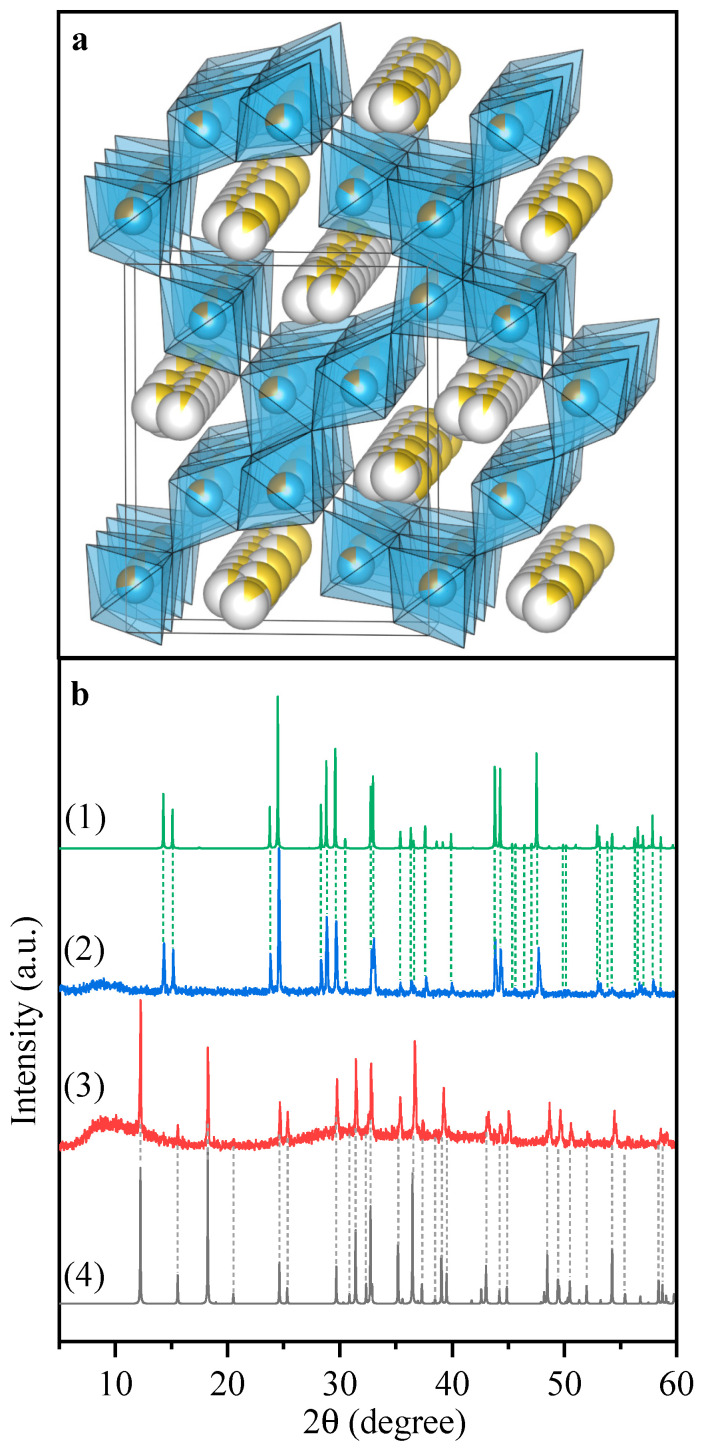
(**a**) Structure of Na_0.9_Fe_0.45_Ti_1.55_O_4_ refined from XRD, exhibiting wide intercalation channels along the b axis. (**b**) XRD powder profiles of as-prepared Na_0.9_Fe_0.45_Ti_1.55_O_4_ sample (**3**) and the same sample after calcination in air, Ox-Na_0.9_Fe_0.45_Ti_1.55_O_4_ (**2**). Powder diffraction profiles of non-stoichiometric iron titanate (**4**) and freudenbergite (**1**) structure types were calculated according to crystallographic data from Crystallography Open Database cards No. 00-100-1512 and 00-901-1187, respectively.

The crystal structure of the Na_0.9_Fe_0.45_Ti_1.55_O_4_ sample is attributed to non-stochiometric iron titanate (Figure 2). All peaks on the XRD pattern could be assigned to orthorhombic symmetry, space group *Pnma* (no. 62). The crystal lattice of the material is characterized by wide channels along the *b* axis, favorable for alkali metal cation intercalation (Figure 2a). A phase transition occurs after calcination in air. The obtained phase Ox-Na_0.9_Fe_0.45_Ti_1.55_O_4_ exhibited a freudenbergite structure type with monoclinic symmetry, space group *C12/m1* (no. 12).

**Figure 3:**
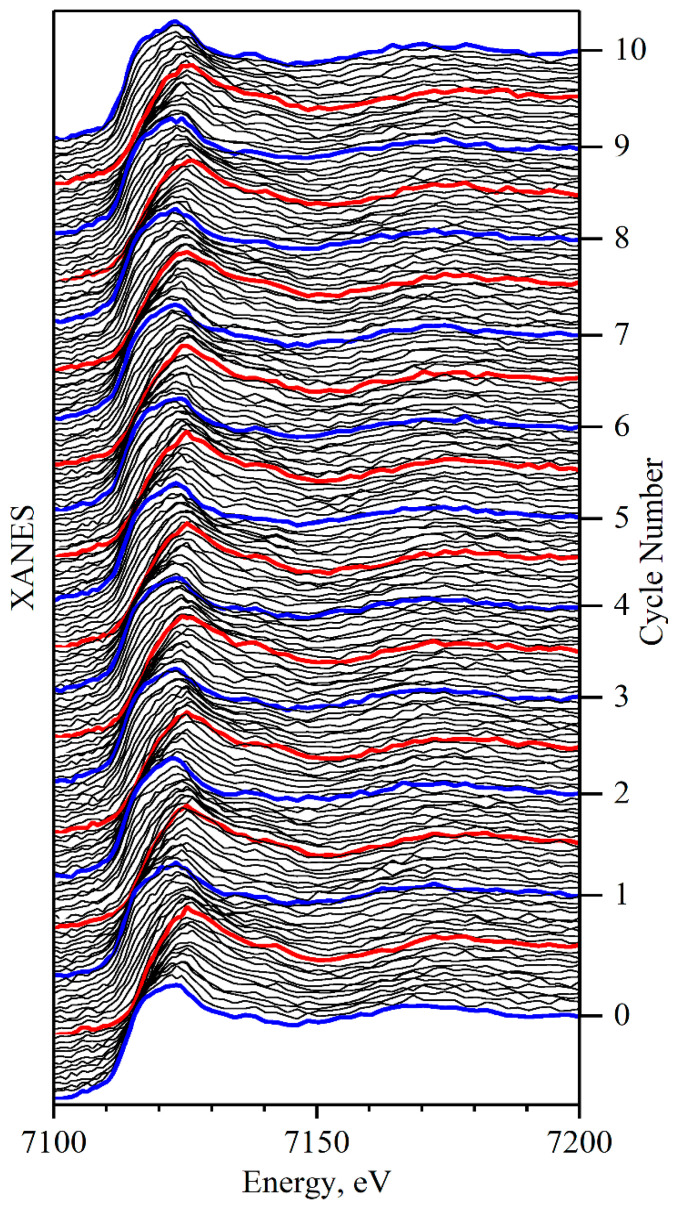
Operando Fe K-XANES spectra for ${\mathrm{Na}}_{x}{\mathrm{Fe}}_{\frac{x}{2}}{\mathrm{Ti}}_{2-\frac{x}{2}}O_{4}$ sample (x = 0.9) during first 10 cycles. Blue and red curves correspond to fully discharged and charged states of the cell, respectively.

Figure 3 shows the Fe K-edge X-ray absorption spectra obtained during the first 10 cycles, where red and blue curves mark the spectra measured for lowest and highest potential, respectively. Each spectrum acquisition took around 25 min. However, in the analysis, we use only part of this energy region, which required ca. 12 min for measuring. Assuming a slow cycling rate, the cell state can be considered stable during single XANES measurement. Prominent changes related to the redox processes can be clearly observed. The chemical shift of the edge position indicates the change in the oxidation state of Fe. The shift of the second absorption maximum around 7170 eV to the higher energies upon charge suggests decreasing interatomic distances. While laboratory X-ray diagnostics lacks a good signal-to-noise ratio in the spectra in comparison to synchrotron data, the tool offers an almost unlimited time range for data acquisition.

**Figure 4:**
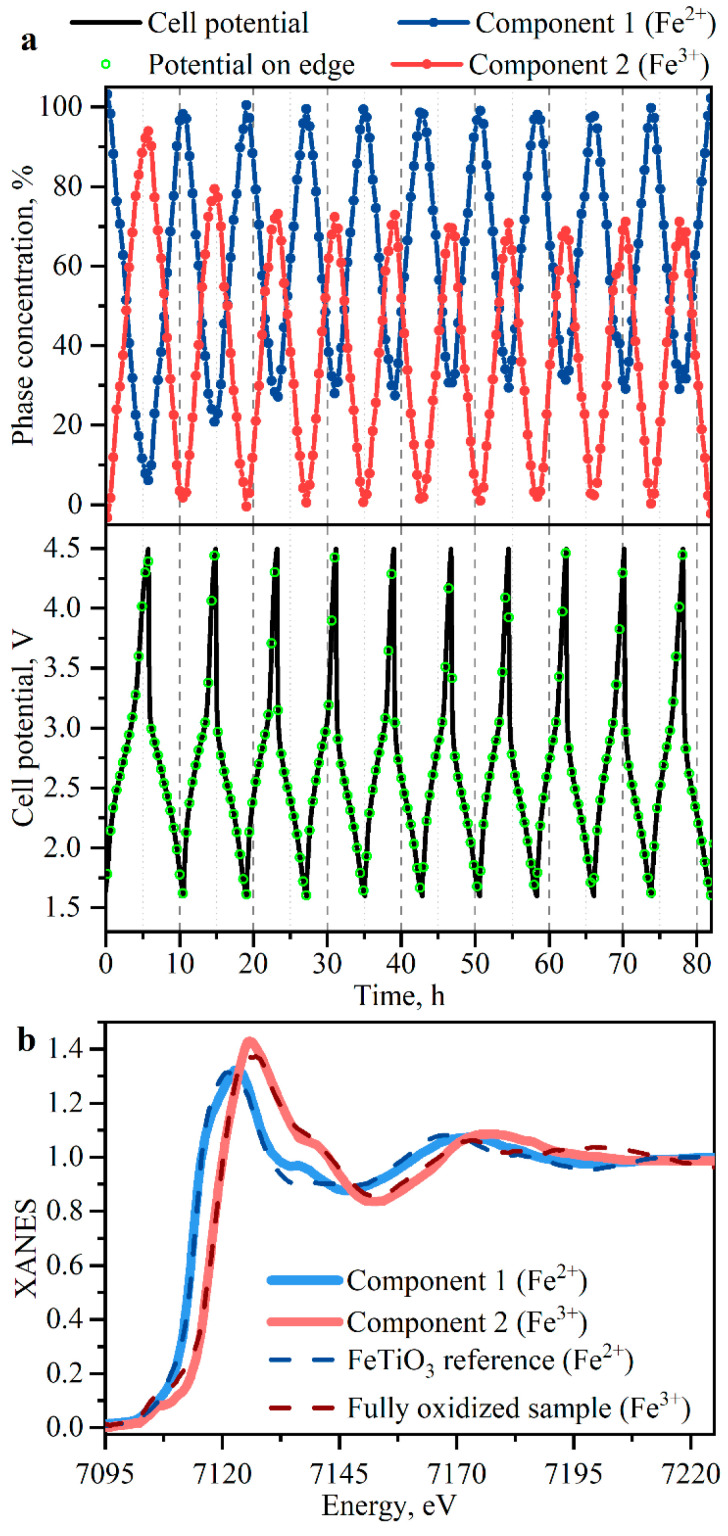
(**a**) Concentrations of the principal components extracted upon PCA of the first 10 cycles and the cell potential plotted on the same time scale. Green circles correspond to the Fe K-edge position in each individual XANES spectrum; (**b**) Spectra of the principal components after target transformation, compared to the experimental spectra of Fe^2+^ and Fe^3+^ reference compounds.

In total, 200 spectra were collected during 10 consecutive cycles over 85 h. Since the XAS spectrum of a multiphase bulk compound is a mere superposition of XAS spectra for each phase, one can use principal component analysis (PCA) to mathematically decompose a series of spectra to extract the pure spectra of involved phases. In this case, we used PCA to extract the spectra of pure phases forming in the electrochemical process and corresponding concentrations. All spectra were interpolated to a single energy range. In the first step, singular value decomposition was applied to extract a given number of abstract components from the series of spectra. Two abstract spectra in our case were further converted to chemically relevant information using target transformation. The following physical constraints were assumed: spectra should be normalized, the values of concentrations should be positive, and at least one of the components under consideration should represent either the fully reduced or fully oxidized phase of the material. In this way, 2 components were successfully identified, as shown in Figure 4.

Figure 4 shows the spectra of the pure phases extracted from PCA compared to the experimental reference spectra of Fe^2+^ and Fe^3+^ compounds. The component corresponding to an Fe^2+^ phase shows good agreement with the FeTiO_3_ reference in terms of absorption edge position and overall profile of the spectrum. The second component can be ascribed to an Fe^3+^ reference compound represented by Ox-Na_0.9_Fe_0.45_Ti_1.55_O_4_, which is basically fully oxidized Na_0.9_Fe_0.45_Ti_1.55_O_4_ sample. Thus, the spectrum of the second component corresponds to electrochemically oxidized Fe^3+^ ions.

The top panel of Figure 4 shows the concentrations of phases corresponding to Fe^2+^ and Fe^3+^ components as a function of time compared to the cell voltage during the first 10 cycles. The material exhibits a single steep plateau on both charge and discharge, while the Fe oxidation state evenly changes across the whole voltage range. The Fe^2+^/Fe^3+^ conversion rate slowly decreases during the first 3 cycles and then stabilizes. This result is consistent with the behavior observed in electrochemistry, where specific capacity on the discharge drops from 70 mAh in the first cycle to an average value of 55 mAh during the fourth and consecutive cycles (Figure S3 in SI). Such behavior may be attributed to the partial collapse of the cathode material structure (intercalation channels, in particular) upon substitution of Na ions to the Li ions since Na_0.9_Fe_0.45_Ti_1.55_O_4_ was cycled vs. Li with Li-ionic electrolyte. 

**Figure 5:**
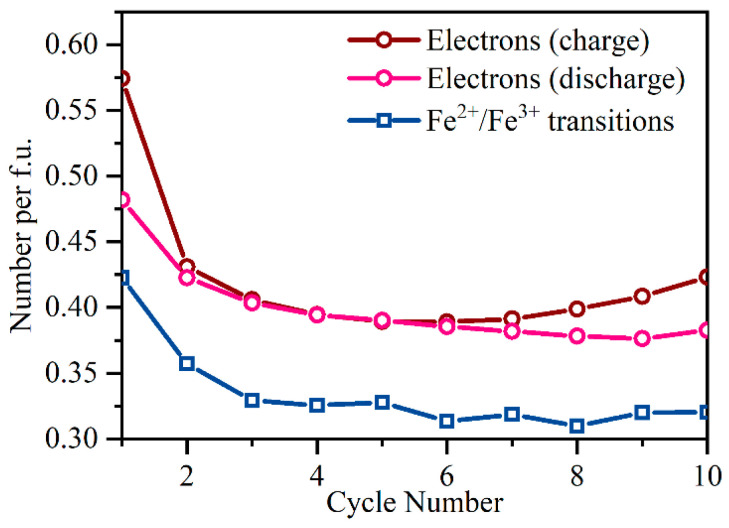
The number of electrons per formula unit transferred through the cell upon charge and discharge as derived from experimental electrochemistry data. The blue bottom curve shows the number of Fe^2+^/Fe^3+^ redox transitions per formula unit obtained from operando XAS as a function of cycle number.

In Figure 5, we quantitatively compare the number of electrons transferred through the cell during discharge with the number of Fe^2+^/Fe^3+^ transitions (both values are per formula unit). Despite sharing the same trend, the number of electrons is 20% higher than the number of redox-active Fe ions in every cycle. This means that 20% of the reversible capacity arises from the electrons participating in another reversible redox reaction, which is different from Fe^2+^/Fe^3+^ transition. The reversible capacity of Li- and Na-ion batteries often is not limited to d-metal cation redox alone, but a notable part of it may be contributed by anion (usually oxygen) [34]. Reversible oxygen redox was reported previously for structures with 4d and 5d elements, such as Li_3_IrO_4_ Li-rich layered rocksalt [35]. However, in high-energy lithium- and manganese-rich (LMR) layered materials, a similar effect may induce a severe capacity loss relative to the initial cycle. The observed reversibility of oxygen redox phenomena is limited to a certain cut-off potential. Beyond this point, O_2_ release from the structure may occur, accompanied by continuous reduction of transition metal cation, activating lower-voltage redox couples and causing microstructural defects [36]. This behavior was reported for Li_4_FeSbO_6_ lithium-rich layered oxide. Unusual Fe oxidation from +3 to +4, and limited reversibility of the oxygenated species, was directly demonstrated during the charge up to 4.2 V, while an oxygen release process occurred upon further charge to 5.0 V [37].

**Figure 6:**
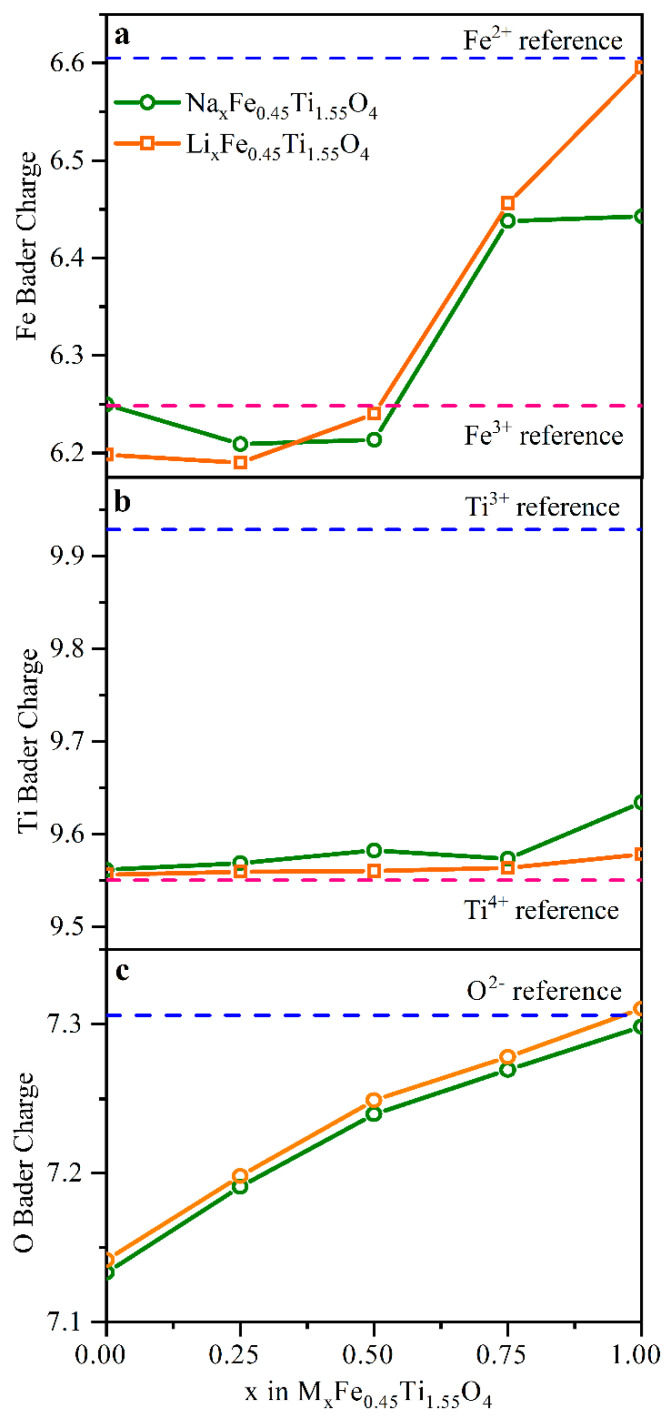
Bader charge analysis for the two sets of M_x_Fe_0.45_Ti_1.55_O_4_ (x = 0, 0.25, 0.5, 0.75 and 1) compositions with Li and Na as M. The values for each atom, namely Fe, Ti and O (panels **a**, **b** and **c**, respectively), were averaged over non-equivalent atomic positions. Reference charge values (dashed lines) were obtained from calculations for reference compounds with different Fe and Ti oxidation states.

To address the origin of additional capacity and validate the possibility of oxygen redox in Na_0.9_Fe_0.45_Ti_1.55_O_4_, a series of ab initio DFT calculations were performed. The voltage range applied for operando cycling covered the sodium stoichiometry range between x = 0.55 and x = 0.9. The geometry optimization and Bader charges were computed for Li_x_Fe_0.45_Ti_1.55_O_4_ and Na_x_Fe_0.45_Ti_1.55_O_4_ in a full range of alkali metals stoichiometry implying x = 0, 0.25, 0.5, 0.75, and 1 compositions. Figure 6 shows the average Bader charge for each type of atom in the cell. Bader charges are topological quantities that cannot be directly compared to the formal oxidation states of elements. Therefore, Table 1 shows Bader charges calculated for reference compounds with a known oxidation state. This comparison makes it possible to assign formal topological charges to the chemically relevant oxidation states. For both Na and Li alkali metals in the lattice, we observed the oxygen redox activity apart from Fe^2+^/Fe^3+^ redox, while Ti atoms did not participate in the redox reaction upon Li cycling. The calculations predict a notable difference in Fe oxidation state for the fully discharged Li and Na compounds (the last point for x = 1 in panel a). In the stoichiometric sample Na_0.9_Fe_0.45_Ti_1.55_O_4_, the iron oxidation state was Fe^2 + δ^ due to partial reduction of Ti atoms. However, Li_0.9_Fe_0.45_Ti_1.55_O_4_ contains iron in an Fe^2+^ charge state. This effect is confirmed by the Fe K-edge XANES (see Figure S4 in SI). The energy position of the absorption edge for the as-prepared sample (Na_0.9_Fe_0.45_Ti_1.55_O_4_) is shifted with respect to the fully discharged sample at subsequent cycles (Li_0.9_Fe_0.45_Ti_1.55_O_4_ stoichiometry). Figure 6 shows that for higher cell potential when Na/Li stoichiometry is below 0.45, the oxygen atoms alone compensate the charge neutrality in the cell. However, this process is unfavorable for the high-voltage region, where molecular oxygen can be released.

To the best of our knowledge, only one compound with pure Fe^2+^ content in a Na_2_O–“FeO”–TiO_2_ system has previously been reported. [38]. The good properties of the studied ${\mathrm{Na}}_{0.9}{\mathrm{Fe}}_{0.45}{\mathrm{Ti}}_{1.55}O_{4}$ material in Li cycling make this material also promising for Na-ion batteries. One of the advantages of Fe^2+^ with respect to Fe^3+^ analogs is lower Fe^2+^/Fe^3+^ redox potential compared to Fe^3+^/Fe^4+^ (−0.77 V vs. 3.4 V) [39]. The higher voltage could damage both electrolyte and electrode, dramatically reducing cycling stability. The number of Na+ ions, which could be safely extracted without phase transition or collapse of the structure in the case of the Fe^2+^ compound, is also higher than for Fe^3+^ analogs.

The cycling behavior of the material was assessed via X-ray absorption spectroscopy. While synchrotron facilities provide the best capabilities in terms of data quality and time resolution, they cannot support the existing huge demand for XAS studies, and much of the routine work does not require an extremely high photon flux. Hence, the development of advanced laboratory tools, including both operando cells and X-ray spectrometers, is complementary to the mega-scale research facilities [40]. The rapid development of X-ray focusing optics and detection systems opens perspectives for new designs of efficient laboratory XAS tools [41,42]. On the other hand, mathematical tools such as principal component analysis improve the quality of quantitative analysis, even for noisy data.

However, the soft X-ray energy region represents a challenge for operando studies, even at synchrotron radiation sources [43,44,45]. Therefore, the use of DFT theoretical approaches is a powerful addition to laboratory experimental studies [46]. We were not able to address the electrochemical behavior of O and Ti atoms in the material using the current experimental setup; however, DFT Bader charge analysis provided the required insight, being complementary to the XAS data on Fe charge state.

## 4. Conclusions

The present work reports operando characterization of the novel sodium-iron-titanate-based Na-ion cathode material ${\mathrm{Na}}_{0.9}{\mathrm{Fe}}_{0.45}{\mathrm{Ti}}_{1.55}O_{4}$. The material was studied during cycling as a cathode in Li-ion half-cell by means of Fe K-edge operando X-ray absorption spectroscopy. Two-hundred spectra obtained over 85 h of electrochemical cycling demonstrate reversible redox reaction and local atomic structure changes for Fe sites during the first 10 cycles. PCA of these data enabled quantitative analysis of the Fe^2+^/Fe^3+^ ratio at each point of the electrochemical process. Both the specific capacity and conversion rate decreased slightly from the first to the fourth cycle and then remained constant. A possible reason for this behavior is the electrochemical substitution of Na by Li in the channels of the cathode material and associated with partial blocking of the ionic shuttle. We determined that the number of electrons transferred during discharge was 20% greater than the number of Fe^2+^/Fe^3+^ transitions derived from PCA of XANES data. Therefore, about 20% of reversible capacity originated from redox reaction other than Fe^2+^/Fe^3+^ transition. DFT calculations and Bader analysis predict the anionic redox process associated with oxygen atoms in the lattice, which accompanies iron charge changes and dominates at high voltages when x < 0.45 in ${\mathrm{Na}}_{x}{\mathrm{Fe}}_{0.45}{\mathrm{Ti}}_{1.55}O_{4}$.

Our work opens new perspectives for the high-throughput screening of novel cathode materials. We demonstrated that laboratory-level equipment can be applied in operando conditions to monitor the charge state of 3d metal ions upon cycling. Quantitative analysis of the spectroscopy data powered by principal component analysis provides the fraction of 3d metals that undergo redox reaction. This quantity is further compared to the number of electrons transferred through the cell and the electrochemically active phase is verified. Experimental data are used as a benchmark for DFT calculations. When the theoretical model reproduces the observed quantities, it can be extended to a range of parameters not accessible by the experimental setup. In this way, we studied the performance of the ${\mathrm{Na}}_{x}{\mathrm{Fe}}_{0.45}{\mathrm{Ti}}_{1.55}O_{4}$ material in the whole range of sodium stoichiometry x = 0…1.

## Figures and Tables

**Table 1:** Averaged DFT Bader charge values for M_x_Fe_0.45_Ti_1.55_O_4_ (where M = Li, Na) and reference compounds with known oxidation state (NaFeO_2_, FeTiO_3_, Ti_2_O_3_).

Species		Na_x_Fe_0.45_Ti_1.55_O_4_					Li_x_Fe_0.45_Ti_1.55_O_4_					Reference Values	
	X	0	0.25	0.5	0.75	1	0	0.25	0.5	0.75	1		
Ti		9.56	9.57	9.58	9.57	9.63	9.56	9.56	9.56	9.56	9.59	Ti^3+^	9.93
Fe		6.25	6.21	6.21	6.44	6.44	6.20	6.19	6.24	6.46	6.60	Ti^4+^	9.55
O		7.13	7.19	7.24	7.27	7.29	7.14	7.20	7.25	7.28	7.31	Fe^2+^	6.61
												Fe^3+^	6.25
												O^2−^	7.31

## Data Availability

The data presented in this study are available on request from the corresponding author. This data is not publicly available due to excessive size and complexity of structuring.

## Supplementary Materials

The following are available online at https://www.mdpi.com/2079-4991/11/1/156/s1, Figure S1: (a) Cell installed into the Rigaku R-XAS Looper laboratory spectrometer and connected to galvanostat. (b) Rigaku R-XAS Looper laboratory spectrometer with additional equipment for operando experiment in front. Figure S2: The first 10 charge–discharge cycles for Na_0.9_Fe_0.45_Ti_1.55_O_4_ sample cycled in Li-ion half-cell in the 1.6 to 4.5 V range with a current of C/20 mA (where C is the theoretical cell capacity). Figure S3: Experimental Fe K-XANES spectrum for as-prepared Na_0.9_Fe_0.45_Ti_1.55_O_4_ sample compared to the spectra of Fe^2+^ (FeTiO_3_) and Fe^3+^ (Ox-Na_0.9_Fe_0.45_Ti_1.55_O_4_) references and its reconstruction with components extracted from PCA. Table S1: Raw Bader charge values obtained from DFT calculations for M_x_Fe_0.45_Ti_1.55_O_4_.
