# Supplementary material for: Laboratory Operando XAS Study of Sodium Iron Titanite Cathode in the Li-Ion Half-Cell
Source: Nanomaterials (Basel). 2021 Jan 9;11(1):156. doi: 10.3390/nano11010156 (PMC7826646; doi:10.3390/nano11010156)
Supplement: Supplementary file 1 [file nanomaterials-11-00156-s001.pdf]

Supplementary information for

Laboratory Operando XAS Study of Sodium Iron Titanite Cathode in the Li-Ion Half-Cell

Victor Shapovalov [1,*], Alexander Guda [1], Vera Butova [1], Igor Shukaev [2] and Alexander Soldatov [1,*]

[1] The Smart Materials Research Institute, Southern Federal University, 178/24 A. Sladkova Street, 344090 Rostov-on-Don, Russia; guda@sfedu.ru (A.G.); vbutova@sfedu.ru (V.B.)
[2] Department of Chemistry, Southern Federal University, 7 Zorge Street, 344090 Rostov-on-Don, Russia; ishukaev@sfedu.ru
* Correspondence: soldatov@sfedu.ru (A.S.); viks@sfedu.ru (V.S.)

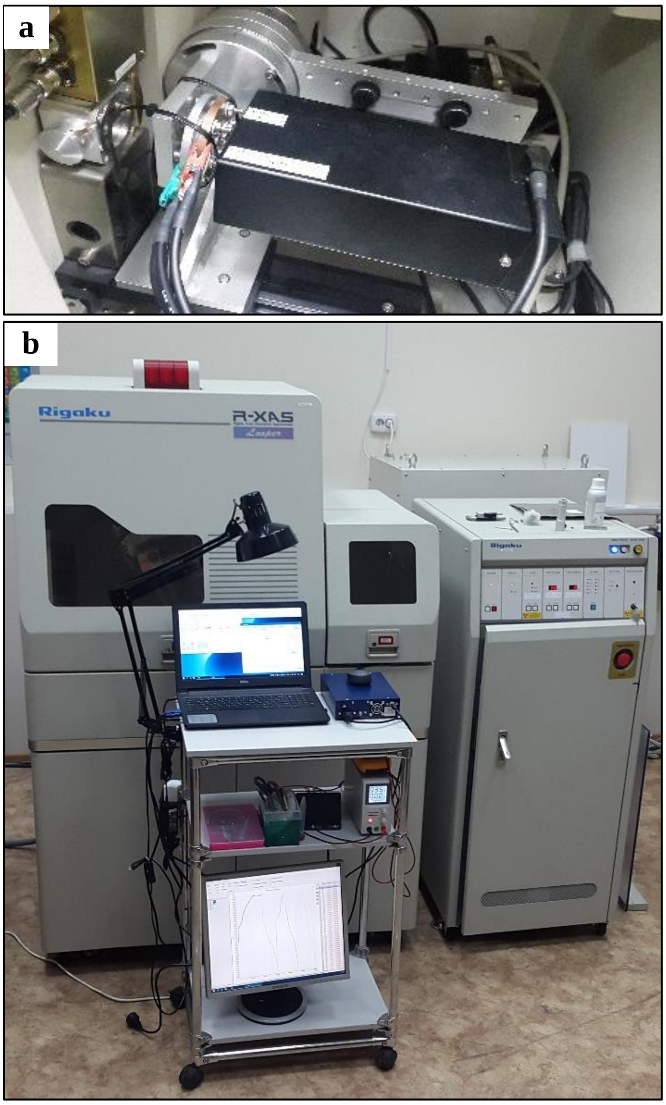

Figure S1. (a) Cell installed into the Rigaku R-XAS Looper laboratory spectrometer and connected to galvanostat. (b) Rigaku R-XAS Looper laboratory spectrometer with additional equipment for operando experiment in front. [1]

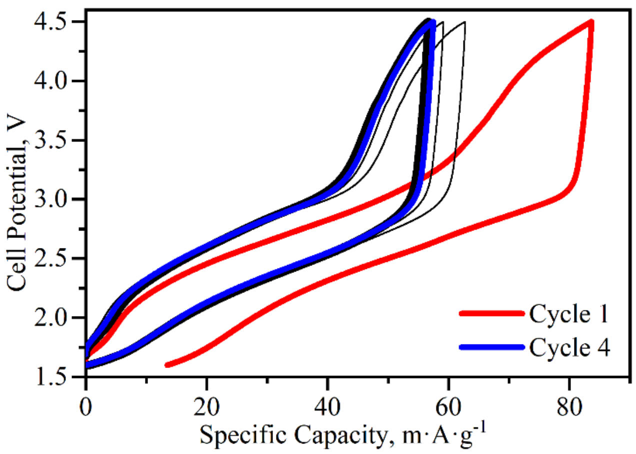

Figure S2. The first 10 charge-discharge cycles for $Na_{0.9}Fe_{0.45}Ti_{1.55}O_4$ sample cycled in Li-ion half-cell in the 1.6 to 4.5 V range with a current of C/20 mA (where C is the theoretical cell capacity).

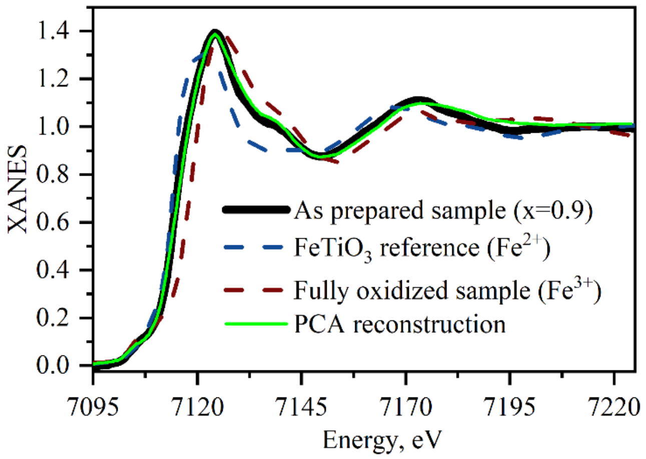

Figure S3. Experimental Fe K-XANES spectrum for as-prepared $Na_{0.9}Fe_{0.45}Ti_{1.55}O_4$ sample compared to the spectra of Fe^{2+} ($FeTiO_3$) and Fe^{3+} (Ox-$Na_{0.9}Fe_{0.45}Ti_{1.55}O_4$) references and its reconstruction with components extracted from PCA.

Despite the agreement with the spectrum of as-prepared sample is quite poor for both PCA components, it can be reasonably well reproduced by their combination, indicating that at the time of measurements Fe in the as-prepared sample was already oxidized to some extent (Figure S3). Minor differences between the experimental profile and PCA reconstruction might be accounted by possible electrochemical substitution of Na with Li which presumably takes place during cycling and causes the change in local atomic and electronic structure of material.

Table S1. Raw Bader charge values obtained from DFT calculations for $M_xFe_{0.45}Ti_{1.55}O_4$.

Atom \ X	$Na_xFe_{0.45}Ti_{1.55}O_4$					$Li_xFe_{0.45}Ti_{1.55}O_4$				
	0	0.25	0.5	0.75	1	0	0.25	0.5	0.75	1
Ti1	9.560	9.571	9.592	9.572	9.577	9.554	9.558	9.572	9.558	9.559
Ti2	9.559	9.577	9.590	9.571	9.575	9.553	9.565	9.567	9.567	9.558
Ti3	9.560	9.571	9.586	9.577	9.912	9.557	9.569	9.559	9.601	9.570
Ti4	9.565	9.566	9.584	9.581	9.577	9.560	9.560	9.564	9.579	9.568
Ti5	9.558	9.570	9.582	9.573	9.568	9.554	9.569	9.558	9.567	9.567
Ti6	9.561	9.563	9.580	9.565	9.911	9.555	9.559	9.566	9.566	9.561
Ti7	9.568	9.580	9.593	9.577	9.577	9.560	9.562	9.570	9.567	9.568
Ti8	9.563	9.578	9.596	9.583	9.580	9.556	9.565	9.573	9.565	9.573
Ti9	9.573	9.571	9.579	9.582	9.577	9.562	9.559	9.564	9.573	9.570
Ti10	9.557	9.558	9.581	9.571	9.579	9.554	9.554	9.563	9.566	9.570
Ti11	9.563	9.560	9.584	9.569	9.574	9.555	9.557	9.559	9.567	9.563
Ti12	9.555	9.566	9.574	9.572	9.903	9.548	9.565	9.548	9.560	9.549
Ti13	9.558	9.554	9.561	9.555	9.570	9.554	9.543	9.558	9.547	9.573
Ti14	9.564	9.558	9.565	9.573	9.586	9.561	9.545	9.554	9.550	9.560
Ti15	9.556	9.585	9.572	9.586	9.588	9.554	9.582	9.555	9.584	9.564
Ti16	9.561	9.572	9.595	9.579	9.585	9.555	9.552	9.561	9.548	9.568
Ti17	9.561	9.565	9.594	9.561	9.589	9.554	9.544	9.557	9.538	9.568
Ti18	9.556	9.545	9.561	9.554	9.912	9.551	9.536	9.543	9.530	9.539
Ti19	9.562	9.575	9.588	9.566	9.590	9.563	9.574	9.553	9.562	9.553
Ti20	9.561	9.561	9.580	9.573	9.575	9.555	9.557	9.557	9.563	9.563
Ti21	9.570	9.574	9.582	9.576	9.571	9.562	9.559	9.565	9.572	9.904
Ti22	9.560	9.564	9.593	9.569	9.574	9.554	9.559	9.566	9.554	9.572
Ti23	9.558	9.581	9.580	9.585	9.583	9.553	9.551	9.547	9.553	9.568
Ti24	9.559	9.582	9.584	9.585	9.582	9.555	9.579	9.561	9.589	9.557
Fe1	6.206	6.212	6.227	6.653	6.649	6.203	6.202	6.209	6.639	6.647
Fe2	6.442	6.235	6.215	6.209	6.271	6.201	6.197	6.185	6.191	6.658
Fe3	6.195	6.199	6.201	6.652	6.649	6.190	6.187	6.206	6.197	6.654
Fe4	6.203	6.204	6.228	6.228	6.222	6.198	6.177	6.512	6.640	6.653
Fe5	6.341	6.204	6.189	6.227	6.661	6.201	6.187	6.190	6.501	6.654
Fe6	6.213	6.210	6.220	6.653	6.221	6.203	6.206	6.205	6.641	6.651
Fe7	6.204	6.199	6.198	6.651	6.648	6.198	6.180	6.199	6.623	6.635
Fe8	6.196	6.209	6.231	6.232	6.222	6.191	6.185	6.215	6.218	6.209
O1	7.205	7.224	7.262	7.259	7.263	7.207	7.222	7.228	7.240	7.242
O2	7.207	7.215	7.226	7.226	7.268	7.213	7.217	7.228	7.220	7.322
O3	7.177	7.235	7.233	7.252	7.278	7.199	7.209	7.235	7.240	7.332
O4	7.247	7.252	7.248	7.300	7.308	7.252	7.256	7.276	7.279	7.293
O5	7.189	7.200	7.191	7.242	7.255	7.193	7.207	7.223	7.244	7.240
O6	7.249	7.285	7.287	7.293	7.305	7.249	7.258	7.277	7.281	7.284
O7	7.187	7.202	7.254	7.252	7.273	7.190	7.201	7.229	7.243	7.246
O8	7.250	7.253	7.255	7.277	7.320	7.254	7.262	7.261	7.291	7.293
O9	7.188	7.193	7.192	7.238	7.257	7.190	7.203	7.218	7.243	7.250
O10	7.247	7.259	7.261	7.275	7.320	7.249	7.263	7.261	7.287	7.288
O11	7.189	7.229	7.254	7.256	7.268	7.192	7.207	7.201	7.205	7.237
O12	7.252	7.266	7.284	7.293	7.313	7.254	7.264	7.276	7.273	7.294
O13	7.208	7.246	7.262	7.247	7.248	7.213	7.221	7.235	7.232	7.286
O14	7.209	7.214	7.253	7.245	7.251	7.207	7.212	7.247	7.231	7.244

O15	7.248	7.261	7.261	7.281	7.312	7.252	7.265	7.269	7.288	7.294
O16	7.162	7.194	7.189	7.266	7.253	7.198	7.205	7.216	7.238	7.251
O17	7.073	7.159	7.166	7.287	7.309	7.086	7.236	7.284	7.272	7.358
O18	7.072	7.150	7.226	7.228	7.325	7.079	7.213	7.183	7.285	7.318
O19	6.756	7.032	7.227	7.238	7.266	6.852	6.927	7.299	7.312	7.376
O20	6.892	7.022	7.158	7.223	7.254	6.920	7.159	7.232	7.314	7.368
O21	6.913	7.151	7.165	7.240	7.287	6.925	7.220	7.214	7.337	7.360
O22	6.909	6.977	7.234	7.238	7.274	6.902	6.930	7.290	7.313	7.365
O23	6.904	7.046	7.205	7.219	7.291	6.893	7.134	7.250	7.280	7.344
O24	6.936	7.075	7.137	7.255	7.275	6.947	7.182	7.268	7.281	7.360
O25	6.901	7.030	7.217	7.268	7.274	6.901	7.125	7.293	7.282	7.404
O26	6.921	7.139	7.224	7.221	7.279	6.925	7.194	7.144	7.263	7.389
O27	6.936	7.156	7.221	7.243	7.272	6.946	7.221	7.224	7.323	7.353
O28	6.887	6.947	7.075	7.225	7.277	6.894	6.928	7.241	7.323	7.377
O29	6.828	6.937	7.126	7.253	7.304	6.851	6.895	7.246	7.227	7.396
O30	7.072	7.188	7.249	7.268	7.320	7.077	7.252	7.245	7.346	7.342
O31	7.079	7.188	7.261	7.276	7.339	7.085	7.238	7.281	7.325	7.310
O32	6.899	7.010	7.125	7.187	7.258	6.918	7.136	7.218	7.163	7.333
O33	7.177	7.199	7.251	7.301	7.344	7.199	7.212	7.251	7.264	7.300
O34	7.128	7.173	7.230	7.280	7.330	7.141	7.195	7.187	7.320	7.338
O35	7.075	7.151	7.217	7.239	7.288	7.067	7.085	7.190	7.270	7.310
O36	7.072	7.128	7.211	7.257	7.276	7.088	7.120	7.264	7.223	7.293
O37	7.084	7.217	7.218	7.258	7.304	7.091	7.164	7.252	7.235	7.254
O38	7.081	7.159	7.278	7.274	7.334	7.080	7.079	7.202	7.236	7.238
O39	7.090	7.089	7.216	7.242	7.291	7.088	7.136	7.195	7.294	7.246
O40	7.094	7.154	7.140	7.245	7.275	7.100	7.165	7.174	7.271	7.235
O41	7.077	7.110	7.153	7.243	7.270	7.079	7.141	7.167	7.281	7.296
O42	7.088	7.176	7.213	7.247	7.293	7.092	7.169	7.137	7.291	7.257
O43	7.092	7.228	7.271	7.260	7.333	7.098	7.169	7.167	7.214	7.258
O44	7.084	7.098	7.216	7.265	7.301	7.089	7.084	7.223	7.214	7.296
O45	7.053	7.150	7.214	7.270	7.289	7.067	7.071	7.222	7.165	7.296
O46	7.129	7.224	7.284	7.296	7.310	7.141	7.179	7.234	7.297	7.340
O47	7.190	7.207	7.253	7.276	7.330	7.198	7.212	7.274	7.309	7.370
O48	7.067	7.108	7.145	7.241	7.294	7.087	7.149	7.179	7.231	7.290
O49	7.287	7.291	7.296	7.306	7.312	7.291	7.290	7.301	7.312	7.314
O50	7.285	7.289	7.303	7.298	7.329	7.287	7.286	7.298	7.306	7.310
O51	7.252	7.287	7.308	7.305	7.320	7.274	7.276	7.297	7.305	7.316
O52	7.314	7.321	7.335	7.334	7.345	7.322	7.322	7.330	7.330	7.339
O53	7.270	7.292	7.289	7.293	7.303	7.275	7.287	7.293	7.301	7.306
O54	7.284	7.295	7.305	7.312	7.307	7.289	7.290	7.292	7.313	7.313
O55	7.268	7.265	7.293	7.301	7.310	7.269	7.270	7.294	7.308	7.306
O56	7.318	7.315	7.320	7.335	7.346	7.322	7.320	7.326	7.336	7.335
O57	7.268	7.266	7.291	7.293	7.303	7.269	7.267	7.291	7.300	7.303
O58	7.282	7.286	7.293	7.308	7.315	7.289	7.289	7.290	7.315	7.311
O59	7.270	7.293	7.291	7.300	7.313	7.275	7.289	7.264	7.276	7.325
O60	7.319	7.322	7.333	7.337	7.350	7.323	7.321	7.330	7.332	7.341
O61	7.281	7.295	7.307	7.308	7.318	7.287	7.292	7.301	7.308	7.307
O62	7.289	7.298	7.322	7.306	7.309	7.292	7.293	7.295	7.310	7.318
O63	7.320	7.321	7.335	7.327	7.353	7.321	7.318	7.326	7.349	7.342

O64	7.249	7.269	7.281	7.305	7.300	7.272	7.271	7.290	7.293	7.316

References

1. Shapovalov, V.V., et al., *Laboratory operando Fe and Mn K-edges XANES and Mössbauer studies of the $LiFe_{0.5}Mn_{0.5}PO_4$ cathode material*. Radiation Physics and Chemistry, 2020, *175*: p. 108065.
